# Detection of Colorectal Cancer by Serum and Tissue Protein Profiling: A Prospective Study in a Population at Risk

**DOI:** 10.4137/bmi.s790

**Published:** 2008-07-02

**Authors:** Judith Y.M.N. Engwegen, Annekatrien C.T.M. Depla, Marianne E. Smits, Annemieke Cats, Henriëtte Tuynman, Henk A. van Heukelem, Pleun Snel, Wouter Meuleman, Lodewyk F. Wessels, Jan H.M. Schellens, Jos H. Beijnen

**Affiliations:** 1 Department of Pharmacy and Pharmacology, The Netherlands Cancer Institute/Slotervaart Hospital, Amsterdam, The Netherlands; 2 Department of Gastroenterology and Hepatology, Slotervaart Hospital, Amsterdam, The Netherlands; 3 Department of Gastroenterology and Hepatology, Antoni van Leeuwenhoek Hospital/The Netherlands Cancer Institute, Amsterdam, The Netherlands; 4 Department of Molecular Biology, Antoni van Leeuwenhoek Hospital/The Netherlands Cancer Institute, Amsterdam, The Netherlands; 5 Department of Medical Oncology, The Netherlands Cancer Institute/Antoni van Leeuwenhoek Hospital, Amsterdam, The Netherlands; 6 Utrecht University, Faculty of Science, Department of Pharmaceutical Sciences, Division of Biomedical Analysis, Utrecht, The Netherlands

**Keywords:** biomarkers, colorectal cancer, SELDI-TOF MS, validation

## Abstract

Colorectal cancer (CRC) is the second most common cause of cancer-related death in Europe and its prognosis is largely dependent on stage at diagnosis. Currently, there are no suitable tumour markers for early detection of CRC. In a retrospective study we previously found discriminative CRC serum protein profiles with surface enhanced laser desorption ionisation—time of flight mass spectrometry (SELDI-TOF MS). We now aimed at prospective validation of these profiles. Additionally, we assessed their applicability for follow-up after surgery and investigated tissue protein profiles of patients with CRC and adenomatous polyps (AP). Serum and tissue samples were collected from patients without known malignancy with an indication for colonoscopy and patients with AP and CRC during colonoscopy. Serum samples of controls (CON; n = 359), patients with AP (n = 177) and CRC (n = 73), as well as tissue samples from AP (n = 52) and CRC (n = 47) were analysed as described previously. Peak intensities were compared by non-parametric testing. Discriminative power of differentially expressed proteins was assessed with support vector machines (SVM). We confirmed the decreased serum levels of apolipoprotein C-1 in CRC in the current population. No differences were observed between CON and AP. Apolipoprotein C-I levels did not change significantly within 1 month post-surgery, although a gradual return to normal levels was observed. Several proteins differed between AP and CRC tissue, among which a peak with similar mass as apolipoprotein C-1. This peak was increased in CRC compared to AP. Although we prospectively validated the serum decrease of apolipoprotein C-1 in CRC, serum protein profiles did not yield SVM classifiers with suitable sensitivity and specificity for classification of our patient groups.

## Introduction

Colorectal cancer (CRC) accounts for about 10% of cancer deaths annually and is thereby is the second most common cause of cancer-related death in both men and women in Europe (1). Upon diagnosis, most patients already have developed locally advanced or metastasised disease. More will develop metastasis during follow-up. Though, when diagnosed and treated early, the overall 5-year survival rate is around 90%. Unfortunately, there are currently no suitable tumour markers for early diagnosis of CRC. Non-invasive diagnostic methods that could be suitable for screening patients, such as measurement of serum carcinoembryonic antigen (CEA) levels, faecal occult blood testing and faecal DNA analysis, all have low sensitivities and/or specificities (2–5).

Recently developed technologies like genomic and proteomic profiling provide new opportunities to search for diagnostic biomarkers. Microarray analyses have yielded highly predictive prognostic profiles for e.g. breast cancer (6). However, genomic analyses depend on the availability of tissue material to assess acquired genetic changes and are thus less suitable for screening large populations or follow-up of patients after surgery. In contrast, proteomic profiling can be done in easily-accessible body fluids like serum, which can be assessed before, during and after (treatment for) CRC. As tissue is constantly perfused by the blood, (tumour) tissue-originating proteins, but also tissue-processed endogenous proteins are represented in the blood and can reflect the actual state of an individual’s health.

Several techniques can be used for protein profiling (for an overview see e.g. (7)). To assess large sample groups, a high throughput method that does not need much pre-analytical sample clean-up is preferable. We and others have previously searched for CRC serum protein profiles with surface-enhanced laser desorption ionisation-time of flight mass spectrometry (SELDI-TOF MS) (8–13). Although much debate has surrounded this technology, several discriminating proteins in these studies overlapped, indicating inter-laboratory reproducibility and validity. However, in these studies most CRC patients had advanced disease and were compared to healthy controls. As the ultimate goal is to use protein profiling for early diagnosis, possibly even in pre-cancerous stages, prospective studies are needed in a more heterogeneous population of patients at risk. Furthermore, for protein profiles to be suitable for follow-up of CRC patients, their levels should be reflective of response to treatment or relapse.

Comparison of tissue protein profiles can give more insight into the pathophysiological mechanisms underlying or accompanying CRC. Additionally, comparing tissue protein profiles of different stages along the so-called adenoma-carcinoma sequence can provide knowledge on the extent of transformation that has occurred in the different histologic subtypes of polyps.

In the current study we aimed to prospectively validate our previous CRC serum protein profiles with SELDI-TOF MS in a new population of patients with an indication for colonoscopy. After colonoscopy patients were attributed to a control group, a group with adenomatous polyps or a group with colorectal cancer. Serum protein profiles of these groups were compared for discriminating proteins. Additionally, serum protein profiles of patients with colorectal cancer at baseline were compared to those more than 3 weeks after surgical resection of the tumour. Lastly, tissue protein profiles were acquired from polyp and CRC tissues and compared.

## Materials and Methods

### Patients and samples

Patients above 18 years old with no history of malignancies (curatively treated melanoma and cervix carcinoma excluded) presenting with an indication for colonoscopy at the department of Gastroenterology and Hepatology or presenting for treatment of a colorectal tumour were asked for participation in this study. Patients were included in two hospitals: The Slotervaart Hospital or the Netherlands Cancer Institute/Antoni van Leeuwenhoek Hospital (both in Amsterdam, The Netherlands). The study was approved by the local medical ethics committees of both institutes and informed consent of every patient was obtained.

**Figure 1:**
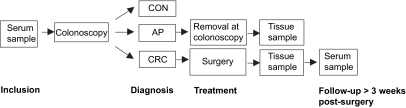
Study set-up.

A serum sample was collected from each participant either before colonoscopy or, when applicable, before treatment of a colorectal tumour (Fig. 1). Following colonoscopy, diagnosis was recorded for each individual. Three groups were defined: control (CON), adenomatous polyps (AP) and colorectal cancer (CRC). A second serum sample was collected from CRC patients at least 3 weeks after surgery if no adjuvant chemotherapy was given. Serum collection was done following a strict protocol in which samples were collected in 9.5-ml BD Vacutainer^®^ SST^™^ tubes (Becton Dickinson, Breda, The Netherlands) and allowed to clot for exactly 30 min at room temperature, after which they were centrifuged at 1500 g for 15 min at room temperature. Samples were then immediately aliquotted and stored at −70 °C.

Tissue samples were collected from patients with adenomatous polyps >0.5 cm or CRC. Tissue was collected dry and tissue sections snap frozen in liquid nitrogen immediately after collection at the department of Pathology. Tissues were stored in liquid nitrogen until analysis.

### Preparation of tissue lysates

Snap frozen tissue sections were disintegrated in deep frozen state by pulverisation with a Micro-dismembranator II (Sartorius AG, Göttingen, Germany) (14). First, tissues were cut into smaller blocks, placed into a pre-cooled shaking flask with a stainless steel ball and then pulverised in three rounds of shaking (55s) and cooling in liquid nitrogen (3 min). Ten mg of the resulting frozen tissue powder was then added to 100 μl of denaturation buffer consisting of 9 M urea, 2% 3-[(3-cholamidopropyl)dimethylammonio]-1-propanesulfonate (CHAPS) and 1% dithiotreitol (DTT) and stored at −70 °C until analysis. For measurement of the protein concentration lysates were thawed on ice, centrifuged at 15000 rpm for 5 min and the supernatant collected for protein profiling. Protein concentration in the supernatants was determined using the 2D-Quant Kit (GE Healthcare, Diegem, Belgium) according to the manufacturer’s instructions.

### Protein profiling

All serum samples were analysed with SELDI-TOF MS (Biorad Laboratories, Hercules, CA, U.S.A.) on CM10 chips as described previously (10). Sample processing was manual, therefore, all samples were randomly attributed to one of nine measurement series before analysis and each series was measured in duplicate on one day. Sample allocation to the chips was randomised, but duplicates were spotted on different chips to take into account inter-chip variability. Pre- and post-surgery sera of CRC patients were assessed similarly in a separate analysis. Tissue lysates were also analysed in duplicate in a separate series using the same procedures as for serum. The amount of lysate applied to the chips was adjusted to the protein concentration in each sample.

Protein chips were read using the PBS-IIC ProteinChip Reader (Biorad Laboratories). Data were collected between 0 and 200 kDa. An average of 105 laser shots per spectrum at laser intensity 140 and detector sensitivity 6 was collected. The focus mass was set to 3000 Da. Settings for tissue analysis were optimised independently, resulting in an average of 105 laser shots per spectrum at intensity 165 and detector sensitivity 6 and a focus mass at 6000 Da. Mass-to-charge (m/z) values were calibrated externally with the all-in-one peptide mixture (Biorad Laboratories).

## Statistics and Bioinformatics

Raw data from all acquired spectra was exported from the Ciphergen ProteinChip Software (Biorad Laboratories) after baseline correction. Normalisation, peak detection and peak clustering were performed using the MASDA R-package (http://bioinformatics.nki.nl). Individual spectra were normalised by centring around zero and dividing by the standard deviation. Peak detection and clustering was performed within a maximum window of 0.3% of the current m/z. All duplicate measurements were averaged and group differences between CON, AP and CRC serum or tissue and between pre- and post-surgery sera were assessed with non-parametric statistical tests corrected for multiple testing using the Benjamini-Hochberg correction. A p-value <0.01 was defined as a statistically significant difference.

Classification models in the form of Support Vector Machines (SVM) were built for each pair wise combination of classes, i.e. CON vs. CRC, CON vs. AP and AP vs. CRC. A radial basis kernel was used for its generally good performance. A double-loop 10-fold cross-validation procedure was used to estimate parameters and to assess model performance (15). Optimisation of the cost parameter *C* and kernel parameter γ was done within the inner loop, whereas the outer loop was used to estimate the performance of a chosen model on an independent test set. To take into account the large prior probability of being classified as CON due to the much larger sample size of this group, performance was estimated using the mean ratio of the true positive and true negative rate. The R-packages e1071 and svmpath were used in the model building process. All other statistical analyses were performed with SPSS version 15.0 (SPSS Inc. Chicago, IL, U.S.A.).

## Results

### Patients and samples

In total, 731 patients with an indication for colonoscopy were asked for participation in this study (Table 1). One hundred and twenty-two patients had to be excluded from serum sample analysis: 41 were found ineligible according to the inclusion criteria, 21 did not give informed consent, 47 did not provide a serum sample for analysis and 13 did not show up for colonoscopy. The total number of patients evaluable for serum protein profiling was 609. The patients excluded for serum analyses did not significantly differ in gender, but were older (64.8 vs. 59.6 years, p = 0.01) and had slightly different indications for colonoscopy and diagnoses compared to evaluable patients. They comprised fewer patients with rectal blood loss (5.9 vs. 13.4%) and more patients with changes in defecation (22.8 vs. 17.0%). Also diagnoses were somewhat different for the excluded patients. There were fewer patients with AP (17.9 vs. 29.0%) and more patients with CRC (18.8 vs. 12.0%) in this group compared to the evaluable patients. Tissue was obtained from 118 individuals, of which 54 CRC. The characteristics of all assessable patients are described in Tables 2 and 3.

### CON, AP and CRC serum protein profiles

We detected 28 significantly different peaks between CON, AP and CRC (Table 4). Most peaks differing between CON and CRC were also significantly different between AP and CRC. No significant differences were observed between CON and AP. Several of the significantly different peaks corresponded to the masses of apolipoprotein C-I and its fragment without the N-terminal threonine and proline (6.6 and 6.4 kDa; doubly charged molecules: 3.3 and 3.2 kDa). Previously identified biomarker candidates at 3.1 and 28 kDa were not detected in the current analyses, and for m/z 4.5 no expression difference was observed. No correlation with age or polyp size was observed for any of the discriminating peaks. All detected peaks were used for model-building with SVM. However, no suitable model was obtained for the discrimination of CON, AP and CRC. The mean 10-fold cross-validation performances of the SVM classifiers for comparison of CON vs. CRC, CON vs. AP and AP vs. CRC were 57.7%, 59.4% and 50.1% respectively.

### Pre- and post surgery serum protein profiles

Pre- and post-surgery serum samples were available from 24 CRC patients (marked in Table 3). Post-surgery samples were drawn 26–201 days after surgery. No significant differences were observed between the pre- and post-surgery serum protein profiles. Sixteen of 24 patients demonstrated an increase for m/z 3315 post-surgery, as well as 14 patients for m/z 6628. Post-surgery peak intensities for these peaks correlated with the time between surgery and the collection of the post-surgery serum sample (r = 0.4, p ⩽ 0.05).

### CON, AP and CRC tissue protein profiles

**Figure 2:**
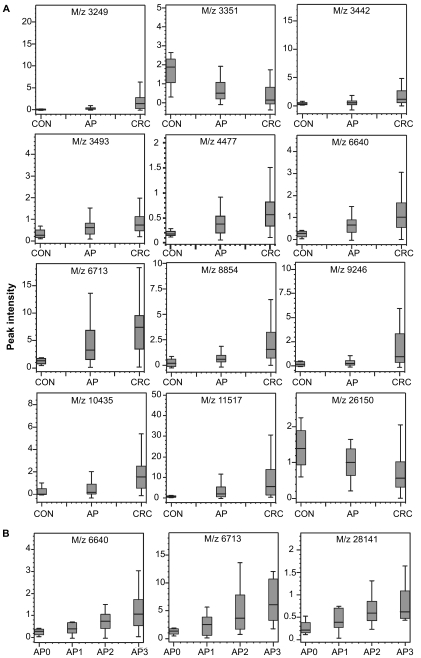
Tissue protein peak intensities in **A)** CON, AP and CRC and **B)** AP0, AP1, AP2, AP3.

**Figure 3:**
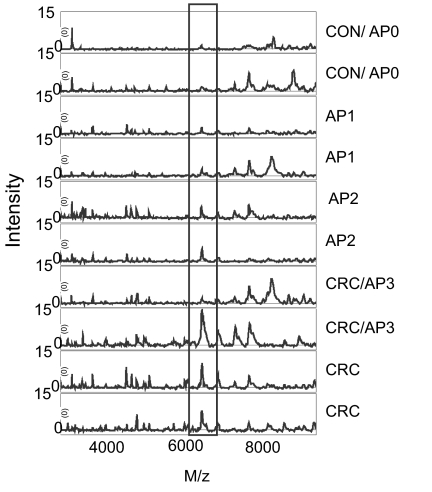
Representative tissue protein profiles. M/z 6713 is shown in the box as an example of a protein differing between CON, AP and CRC.

From 103 patients tissue protein profiles were evaluable (Table 2b). Tissues were classified according to their histology as CON (hyperplastic polyps, AP0), AP (tubular and tubulovillous polyps; AP1 and AP2 respectively) and CRC (tumour tissue and polyps with carcinoma *in situ* (AP3)). We found 31 peaks that were significantly different between tissues (Table 5), many of which showed a gradual increase or decrease going from CON to AP to CRC (Fig. 2A). Peaks with m/z 6.6 kDa were found that were increased in CRC compared to AP and CON and also across the different subtypes of polyps (Fig. 2B). E.g., for m/z 6640 the peak intensities of polyps with carcinoma *in situ* largely overlapped with those for CRC tissue (compare Fig. 2A and B), whereas peak intensities in tubular polyps (AP1) are closer to those of hyperplastic polyps (AP0). However, we were unable to classify patients with sufficiently good sensitivity and specificity based on these tissue protein profiles. Examples of tissue spectra are shown in Figure 3. A correlation with polyp size was observed for peaks 6640 (r = 0.369; p = 0.003), 6713 (r = 0.419; p = 0.001), 11517 (r = 0.382; p = 0.002) and 12705 Da (r = −0.390; p = 0.002).

## Discussion

In the current study we prospectively validated our previous serum protein profiles in CRC patients with advanced disease in a new population of patients with mostly early-stage CRC, adenomatous polyps and controls with an indication for colonoscopy. To our knowledge, this is the first large-scale clinical study in which previously established CRC serum protein profiles are validated in a clinically relevant population of patients with an indication for colonoscopy. Of the 690 eligible patients who were asked to participate in this study, 609 were evaluable for serum analysis (88.3%). Small differences between the evaluable and non-evaluable patients were the older age of the latter group and different indications and diagnoses for colonoscopy. Comparison of latter indicates that patients with the least and most severe diagnosis were most likely to be non-evaluable. That is, patients whose complaints disappeared were more likely to miss colonoscopy, whereas patients with CRC were more likely to give no consent because of their disease. The omission of these extremes in the eligible population may have precluded the detection of more differences between patients and controls. We confirmed the decreased expression of m/z 3.3 and 6.6 kDa, two ions of apolipoprotein C-I, in the current population of CRC patients. Recently we have shown that one of our earlier biomarker candidates, an N-terminal albumin fragment of 3.1 kDa, is a product of proteolysis during storage at −20 °C (16), explaining why we did not observe this peak in this analysis. The lack of an expression difference for peaks at 4472 Da and 28 kDa is likely related to the different patient characteristics or sample handling protocol compared to our previous study. We did not find any significant differences between pre- and post-surgery serum protein profiles. As apolipoprotein C-I is synthesised mainly by the liver and small intestine (17), it is plausible that resection of the tumour does not immediately influence the abundance of this protein. However, we observed a correlation between the post-surgery peak intensities of m/z 3.3 and 6.6 kDa and the time between surgery and serum collection, indicating that apolipoprotein C-I levels do return to normal, but with a time-lag of about 3 months after surgery (data not shown). Also for CEA a time lag of 6 to 12 weeks levels after surgery is common before levels return to normal.

Although we validated the serum decrease of apolipoprotein C-I in this group of patients with early-stage CRC, we could not use the acquired serum or tissue protein profiles as such for correct classification of CON, AP and CRC. We expected that the potential to classify patients by their serum protein profile would be lower in the current population, since we compared patients with early-stage disease to a control group which was essentially not ‘healthy’. Because of this, more background noise was introduced in this analysis. However, an analysis in this population is essential for assessment of the clinical utility of this type of mass spectrometric profiling. Importantly, we could not discern patients with AP from CON, which would be advantageous in establishing which patients need a colonoscopy for removal of AP. Quantitative methods are needed to determine the actual differences in serum levels of apolipoprotein C-I between CON, AP and CRC. Then, suitable cut-off levels can be established for clinical use. Such an approach was taken e.g. by Habermann et al., who used an ELISA for quantitation of complement C3a des-Arg and validated their MS results (13). Although others have suggested serum protein profiles on a different chip surface with good sensitivity and specificity for the discrimination of AP and CRC (9), these results remain to be validated in larger patient groups. Therefore, the current serum protein profiles can as yet not replace endoscopic screening. In addition, regarding the specificity of apolipoprotein C-I for CRC, we must remark that a similar decrease of apolipoprotein C-I or protein peaks with similar mass has been described in several other types of cancer and benign disease (10; 18; 19). Hence, its potential usefulness for CRC seems confined to follow-up of patients.

Besides serum, we have compared polyp and CRC tissue in order to elucidate any sequential protein expression differences occurring during the development from hyperplastic polyps to carcinoma *in situ* and CRC. In addition we compared discriminating serum proteins with those in tissue. We found a protein peak of 6.6 kDa, which might represent apolipoprotein C-I, in tissue samples. Contrary to serum, these peaks exhibited a higher abundance in CRC than in AP and CON. However, serum protein levels do not necessarily reflect the processes occurring in tissue. For example, apolipoprotein A-I can be decreased in serum due to less synthesis in the liver, but increased in tissue due to local synthesis (10; 20). A gradual increase of the 6.6-kDa peak and others was obvious looking only at the different histologic subtypes of polyps, indicating that these subtypes indeed reflect the extent of transformation of these polyps. Also, a relationship between peak intensity and polyp size was observed for some peaks, which indicates that polyp size could be a surrogate measure for the extent of malignant transformation. Whether hyperplastic polyps are in fact pre-malignant stages of CRC, as is suggested by the hyperplastic polyposis syndrome (21), and not innocent polyps, remains to be established by direct comparison of hyperplastic polyp and normal tissue protein profiles. Normal tissue was not analysed in the current study, as we did not attempt to find diagnostic tissue proteins, because of the limited utility of tissue as a matrix for screening purposes due to the need for colonoscopy.

Several reports have described the increase of α-defensin 1–3 levels in CRC tissue (22; 23). In the current study we also found peaks with masses corresponding to these proteins, namely m/z 3442, 3375 and 3493. Only the first peak was also found increased in CRC compared to AP and CON in our study. In the pair wise comparison of CON and CRC, we found m/z 3493 borderline significantly increased in CRC (p = 0.019). The facts that we did not compare normal mucosa to CRC and used whole tumour tissue instead of microdissected tumour cells are likely the cause of the discrepancies with published reports. Whereas others have also reported the presence of these peptides in serum and suggested their potential as serum markers (22; 23), we did not detect them in serum under the current binding conditions. The potential of the α-defensins as diagnostic biomarkers for CRC seems limited, as their serum levels are also increased in other cancers and several benign immunological conditions. Furthermore, our tissue protein profiling results indicate that their expression is only increased in CRC and not yet in polyps, hampering their use for the identification of polyps with malignant potential. Yet, as suggested previously, they may be useful as markers for CRC prognosis and monitoring (24). Unlike Melle et al. (25; 26) we did not observe a significant expression difference between AP and CRC at 10.84 or 12.0 kDa that could correspond to heat shock protein 10 or calgizzarin. This may also be caused by the above-mentioned difference in tissue processing.

Concluding, we validated the decrease of apolipoprotein C-I serum levels in CRC in a large prospective study with SELDI-TOF MS. Yet, quantitative methods for apolipoprotein C-I measurement should be developed and used to establish reliable cut-off values for its clinical use. In addition, we found gradual changes in protein expression along the different stages of the adenoma-carcinoma sequence, which may help discern adenomatous polyps with malignant potential.

## Figures and Tables

**Table 1:** Indications for colonoscopy of patients asked for participation in this study.

Indication for colonoscopy	N = 731 (100%)
Rectal blood loss	98 (13.4%)
Abdominal discomfort	101 (13.8%)
Anaemia	34 (4.7%)
Family history of AP or CRC	136 (18.6%)
Follow-up after previous AP	96 (13.1%)
Altered bowel habits/movements	124 (17.0%)
Family history of AP or CRC and abdominal discomfort	23 (3.1%)
Family history of AP or CRC and rectal blood loss	14 (1.9%)
Suspected malignancy (signs of bowel obstruction, suspect lesion by imaging)	21 (2.9%)
FAP gene mutation carrier	3 (0.4%)
At risk for HNPCC	33 (4.5%)
Confirmed HNPCC mutation carrier	12 (1.6%)
Other/unknown	36 (4.9%)

**Table 2a:** Characteristics of diagnostic groups evaluable for serum protein profiling.

	CON (n = 359)	AP (n = 177)	CRC (n = 73)
**Male sex**	163 (45.4%)	89 (50.3%)	43 (58.9%)
**Mean age (years** ± **SD)**	57.4 ± 13.0	61.0 ± 12.1	67.8 ± 12.0^1^
**Polyp histology**			
Hyperplastic	65		
Tubular		120	
Tubulovillous		57	
Carcinoma in situ			12
**Mean polyp size (mm** ± **SD)**	4 ± 2	8 ± 6	17 ± 6^1^
**Median CEA (**μ**g/l) [range]**	N.A.	N.A.	4.10 [0.2–1338]

1 p > 0.001; CON vs. AP vs. CRC.

N.A. not assessed.

**Table 2b:** Characteristics of diagnostic groups evaluable for tissue protein profiling.

	CON (n = 4)	AP (n = 52)	CRC (n = 47)
**Male sex**	3 (75%)	25 (48.1%)	25 (53.2%)
**Mean age (years** ± **SD)**	58.2 ± 8.43	60.4 ± 11.7	67.3 ± 11.9^1^
**Polyp histology**			
Hyperplastic	4		
Tubular		12	
Tubulovillous		39	
Carcinoma in situ			12
**Mean polyp size (mm** ± **SD)**	10 ± 4	15 ± 6	17 ± 8

1 p = 0.012; CON vs. AP vs. CRC.

**Table 3:** Tumour characteristics of evaluable CRC patients.

Patient ID no	pT	pN	pM	Tumour location	Patient ID no	pT	pN	pM	Tumour location
05070535^1^	1	0	0	Sigmoid	06070187	3	0	0	Sigmoid
05070478^2^	0	X			07020087^1^	2	1	X	Colon ascendens
05100137^1^	2	0	0	Sigmoid	06050316	3	1	X	Rectum
06030371	X	X	1	Rectum	06110117	3	1	0	Colon ascendens
06030418^1^	2	0	0	Rectum	06110151^1^	2	2	X	Rectum
06050222	3	0	0	Rectum	07010024	3	1	X	Rectum
06080016^1^	4	2	1	Sigmoid	07020119^1^	2	0	X	Rectosigmoid
06090095^1^	2	0	X	Sigmoid	06020194	3	1	1	Coecum/colon ascendens
06090301^2^	0	X	X	Rectum	06040207^1^	3	1	X	Coecum
06110347	2	0	X	Rectum	06050009	3	0	X	Colon
06120034	.	.	.	Rectum	06050178^1^	3	1	X	Sigmoid
05100238^2^	0	X	X	Sigmoid	06070077	X	X	1	Rectum
06020328^2^	0	X	X	Sigmoid	06070325	3	0	0	Rectum
06030153^1^	3	0	0	Colon transversum	06120047	4	1	X	Coecum/colon ascendens
06030315	2	1	X	Rectum	07030059	.	.	.	Rectum
06050013	3	0	0	Coecum	07030137^2^	0	X	X	Rectum
06070009^1^	3	0	X	Colon ascendens	07030258	3	0	X	Colon ascendens
06070335	3	1	X	Flexura hepatica	07060033	3	0	X	Colon transversum
06070336	3	0	X	Rectum	07060197	2	1	X	Sigmoid
06090220	3	1	0	Flexura hepatica	05100138^1^	3	0	0	Rectosigmoid
06120213	3	1	X	Sigmoid	06070178	X	X	1	Rectum
07010234	X	X	1	Sigmoid	06100105	2	1	X	Rectum
07010316	3	1	X	Sigmoid	06100115^1^	3	0	1	Rectum
05120313^1,2^	1	0	X	Rectum	07040138	2	0	X	Rectum
05080096^1^	3	0	0	Colon transversum	07050173^1^	3	0	X	Sigmoid
06020162^2^	1	X	X	Sigmoid	07060014	3	0	X	Colon
06030355^2^	1	X	X	Sigmoid	07060037	3	.	.	Rectum
06060392	3	1	X	Sigmoïd	07060149	2	X	X	Sigmoid
06070073^1^	3	0	X	Coecum	07060162	2	0	0	Rectum
07010397^1^	3	2	X	Rectum	06060102^1^	3	0	X	Sigmoid
05090276^1^	X	0	X	Rectum	06080189	4	1	1	Appendix
06010268^2^	0	X	X	Sigmoid	07060161	3	0	X	Rectum
06020195^1^	3	0	0	Coecum	07070078	3	1	X	Rectum
06020339^2^	0	X	X	Rectum	07070079	2	.	X	Rectum
06040166^1^	3	0	0	Colon ascendens	06110038^2^	0	X	X	Rectosigmoid
06040213^1^	4	0	0	Sigmoid	07020194^2^	0	X	X	

pT, pN, pM: pathologically determined tumour stage according to the TNM system.

1 Also used in pre- vs. post-surgery comparison.

2 Carcinoma in situ.

**Table 4:** Significantly different peaks in serum protein profiles. (No significant differences between CON and AP were observed.)

M/z (Da)	Multiple testing-corrected *p*-values		
	CON vs. AP vs. CRC	CON vs. CRC	AP vs. CRC
3215	0.01	0.002	0.001
3314	0.0058	0.002	0.001
3315	0.0045	0.007	0.002
4279	NS	0.002	0.009
4625	NS	0.007	0.001
6427	NS	0.002	0.002
6428	NS	0.002	0.002
6625	0.0039	0.004	0.001
6626	0.0039	0.005	0.002
6634	0.0063	0.006	0.002
7559	NS	NS	0.004
11615	NS	0.007	0.007
11649	0.004	0.004	<0.001
11669	NS	NS	<0.001
13241	NS	NS	0.009
15091	NS	NS	0.001
15102	0.005	0.007	<0.001
15114	0.005	0.004	<0.001
15142	NS	0.007	<0.001
15202	NS	NS	<0.001
15303	0.007	NS	<0.001
15327	NS	NS	0.001
18582	NS	0.008	NS
66202	NS	0.007	NS
66328	NS	0.007	NS
66426	NS	0.007	NS
66520	NS	0.008	NS

NS: not significant.

**Table 5:** Significantly different peaks in tissue protein profiles. (No significant differences between CON and AP or between CON and CRC were observed.)

M/z (Da)	Multiple testing corrected *p*-values	
	CON vs. AP vs. CRC	AP vs. CRC
3249	0.001	<0.001
3351	0.010	0.014
3442	0.001	<0.001
4477	0.005	0.010
4903	0.012	0.004
5358	NS	0.010
6622	0.002	0.001
6640	0.004	0.005
6713	0.004	0.006
7669	0.006	0.002
7955	0.001	<0.001
8568	NS	0.005
8854	0.001	<0.001
9246	0.001	<0.001
10435	0.001	<0.001
11517	0.003	0.005
11649	NS	0.006
12705	NS	0.006
13157	0.002	<0.001
15307	0.005	0.002
16022	NS	0.006
26150	0.005	0.004
31090	0.005	0.002
31922	0.001	<0.001
41879	0.006	0.002

NS: not significant.

## Non-Standard Abbreviations

AP: adenomatous polyps
CON: controls
CRC: colorectal cancer
